# Abrogation of TGF-beta signalling in TAGLN expressing cells recapitulates Pentalogy of Cantrell in the mouse

**DOI:** 10.1038/s41598-018-21948-z

**Published:** 2018-02-26

**Authors:** Bashar Aldeiri, Urmas Roostalu, Alessandra Albertini, Julia Behnsen, Jason Wong, Antonino Morabito, Giulio Cossu

**Affiliations:** 1 Manchester Academic Health Science Centre, Division of cell Matrix Biology and Regenerative Medicine, School of Biological Sciences, Faculty of Biology, Medicine and Health, The University of Manchester, Manchester, UK; 2 Royal Manchester Children’s Hospital, Manchester, UK; 3 Manchester University Hospitals, Wythenshawe Hospital, Manchester, UK; 4 Henry Moseley X-Ray Imaging Facility, The University of Manchester, Manchester, UK; 5 San Raffaele Telethon Institute for Gene Therapy (SR-TIGET), IRCSS, San Raffaele Scientific Institute, Milan, Italy

## Abstract

Pentalogy of Cantrell (PC) is a rare multi-organ congenital anomaly that impedes ventral body wall closure and results in diaphragmatic hernia, intra- and pericardial defects. The underlying cellular and molecular changes that lead to these severe developmental defects have remained unknown largely due to the lack of representative animal models. Here we provide in depth characterization of a mouse model with conditional ablation of TGFβRII in Transgelin (*Tagln*) expressing cells. We show that *Tagln* is transiently expressed in a variety of cells that participate in the embryonic development and patterning of ventral structures. Genetic ablation of TGFβRII in these cells leads to ventral midline closure defect, diaphragmatic hernia, dilated cardiac outflow tract and aberrant cardiac septation, providing a reliable model to study the morphological changes leading to PC. We show that myogenisis in the diaphragm is independent of TGFβ and the diaphragmatic hernia arises from fibroblast-specific migration defect. In the dorsal body wall *Tagln* expression is initiated after the closure process, revealing a remarkable difference between ventral and dorsal body walls development. Our study demonstrates the use of micro-CT scanning to obtain a 3-dimensional high-resolution overview of embryonic anomalies and provides the first mechanistic insight into the development of PC.

## Introduction

Pentalogy of Cantrell (PC) is a rare congenital anomaly with an estimated incidence of 1 in 200,000 live births^1^. It involves the presentation of some or all of the five characteristic congenital anomalies namely; a supra umbilical midline closure defect, *ectopia cordis*, anterior diaphragmatic hernia, cardiac anomalies and failure of formation of the diaphragmatic pericardium^2^. The outcome of infants born with PC is relatively poor. Post-operative mortality reaches 50% and less than a third of the reported cases in the literature were alive at time of follow up^3,4^.

The aetiology behind this complicated congenital anomaly is still largely unknown. Cantrell and colleagues suggested that the varied congenital anomalies seen in the pentad are all of mesodermal origin and possibly take place within the first 18 days of embryonic development^5^. They postulated that failure in the development in a segment of the lateral plate mesoderm leads to defective *septum transversum*, pericardial and intra-cardiac defects. In addition, failure of migration of the mesodermal folds towards the ventral midline results in ventral body wall closure defect^5^. However, up until now this theory has never been evaluated. Similarly, no specific genetic abnormality has been conclusively correlated with PC, even though multiple genetic influences and chromosomal associations with PC have been described^6^. The spectrum of Cantrell has been reported in association with chromosomal anomalies like trisomy 18, trisomy 21, trisomy 13 and Turner syndrome^7,8^. In addition the association between PC and X-linked inheritance (in Goltz-Gorlin syndrome and Xq25-26.1 region) has been established^9,10^. An underlying genetic defect behind the development of this mesenchymal-of-origin group of anomalies is thus likely, yet the cellular mechanisms leading to this anomaly have remained unknown.

In the developing embryo transforming growth factor β (TGFβ) signalling plays a pivotal role in facilitating closure of the embryonic midline^11^. It is also essential for cell homeostasis in general and is particularly crucial for cardiac and vascular development^12,13^. Furthermore, defects in TGFΒ signalling pathways are known to associate with cardiac and midline closure defects^14–19^. However, they can also result in various congenital anomalies including dorsal midline closure defects, cleft palate, lung hypoplasia, craniofacial and limb malformations and urogenital defects^11,16,20–22^. Although all TGFβ morphogens signal via common receptors (TGFβR1/2/3 complex) their expression varies between various cells and tissues, explaining the differences in phenotypes when knocked out in the mouse.

Transgelin (TAGLN, also known as SM22a) is an actin-binding cytoskeletal protein that is expressed in vascular smooth muscle cells^23^. At embryonic stages TAGLN is not a specific VSMC marker and is widely expressed by non-vascular mesenchymal tissues^24^. Moreover, *Tagln* was recently found to label a migratory myofibroblasts cell population that respond to TGFβ signalling^15^. TGFβ is also known to induce Transgelin (*Tagln*) *in vitro* and *in vivo*^25–27^ through SMAD binding to the *Tagln* promoter^28^. Furthermore, TGFβ signalling inactivation in *Tagln* expressing cells leads to ventral body wall closure defect, cardiac and great vessels anomalies^15,17,29^.

We have recently demonstrated that selective removal of *Tgfbr2* from TAGLN expressing cells results in exomphalos and *ectopia cordis*^15^. In addition, previous reports of the *Tagln*-Cre:*Tgfbr2*^flx/flx^ have demonstrated cardiac and major vessels defect that overlap with the spectrum of cardiac anomalies seen in PC^17,29^. We are now reporting an anterior diaphragmatic hernia in this model making the congenital defects in the *Tagln*-Cre:*Tgfbr2*^flx/flx^ mouse model of highly representation of the PC anomaly seen in humans.

## Results

### *Tagln*-Cre:*Tgfbr2* knockout exhibits multiple congenital anomalies

**Figure 1:**
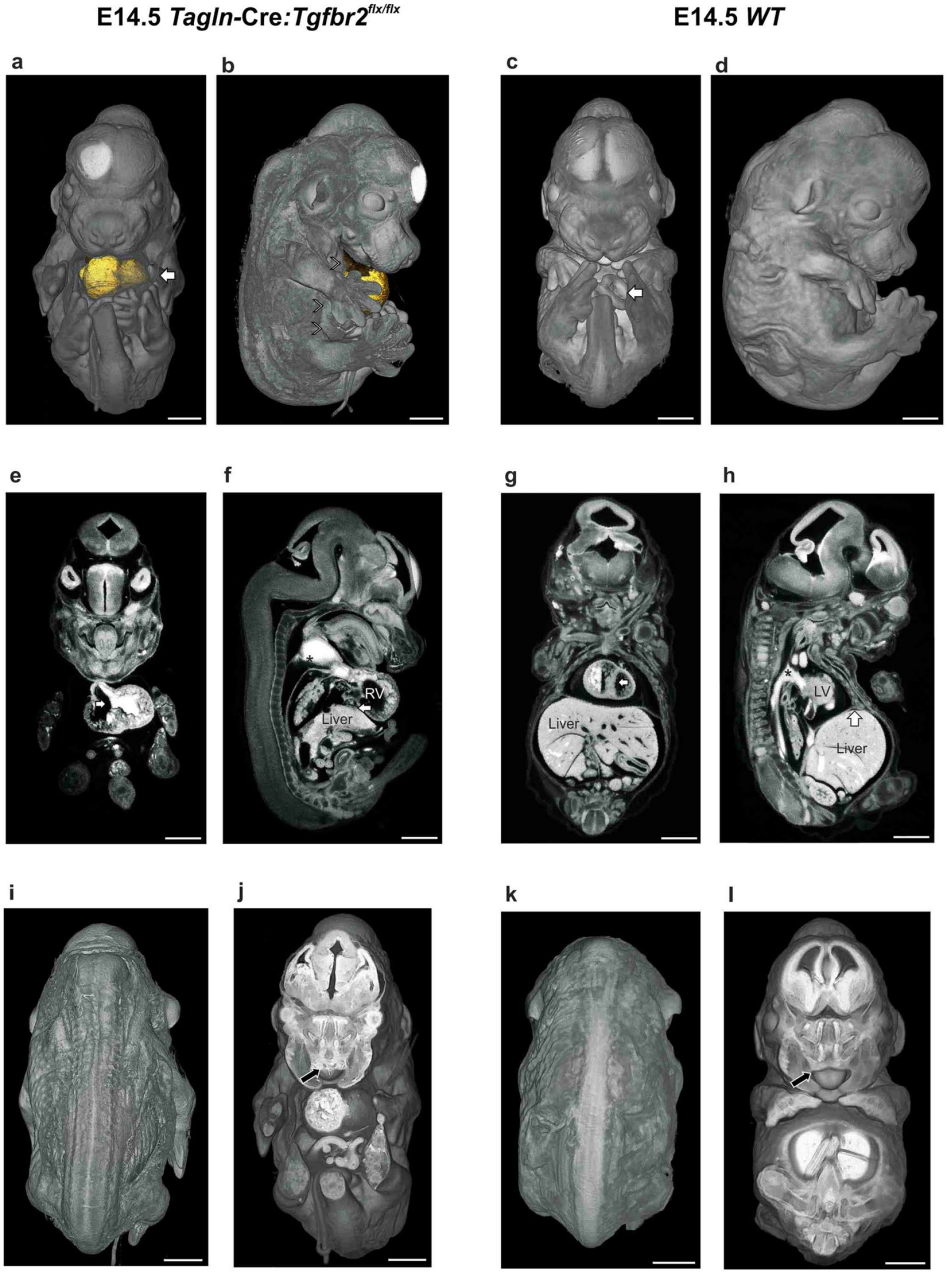
Congenital anomalies in *Tagln*-Cre:*Tgfbr2* ^*flx/flx*^. (**a**–**d**) comparison between the ventral body wall of mutant and *WT* littermates at E14.5 using micro-CT volume rendering and organ segmentation. In the mutant the ventral structures are covered by a thin sac and the umbilical cord (arrow in **a**) is situated at the centre of the exomphalos. The heart (segmented in gold) is seen herniating ventrally on the lateral view and the lateral body wall components (arrowheads in **b**) fail to progress towards the midline. The *WT* littermate show complete closure of the thoracic ventral body wall and only a small physiological umbilical hernia (arrow in **c**) is seen at this developmental stage. (**e–h**) Cardiac and outflow tract anomalies. Ventricular septal defect (arrow in **e**) is visible in the mutant embryos, while the *WT* shows complete ventricular septal closure (arrow in **g**). Dilatation of the right ventricle (RV) and outflow tract (asterisk) compared to the *WT* littermates (f and h respectively). The liver herniates into the chest in the mutant (arrow in **f**). (**i**–**l**) dorsal body wall and palate closure in *Tagln*-Cre:*Tgfbr2* ^*flx/flx*^ are comparable to *WT* littermates. An arrow in (**j** and **l**) indicates the anterior part of the palate. The contrast material is seen filling the cardiac chambers and outflow tract in (**e**–**h**). LV: left ventricle, RV: right ventricle. Scale bars 1,000 µm.

The *Tagln*-Cre:*Tgfbr2* ^*flx/flx*^ model exhibits three main categories of congenital anomalies. The first striking anomaly is the complete failure of ventral body wall closure as we have recently shown^15^. We used here micro-CT scanning to characterise the extent of the defect and to delineate its 3D topography. We found that the knockout embryos develop a large exomphalos and *ectopia cordis*. A thin sac covers the heart and embryonic intestine (Figs 1a,b and S1b,d,e and Movie 1) and the lateral body wall fails to develop beyond the ventral one-half (arrowheads in Fig. 1b and S1e). The wild type littermates show only a small physiological umbilical hernia and the ribs and intercostal muscles reach the midline (Figs 1c,d and S1a,c and Movie. 2). The mutant embryos also show gross cardiac and outflow tract anomalies. Large ventricular septal defect (VSD) is present (Fig. 1e) compared to complete ventricular septal closure in the *WT* littermates (Fig. 1g). Moreover, gross dilatation of the heart and outflow (OF) tract is evident (Figs 1f and S1f) when compared to the *WT* littermates (Fig. 1h). In addition, the central veins showed gross dilatation as well (Fig. S1g,h). Lastly, this model displays an anterior diaphragmatic hernia with the liver herniating into the chest (Fig. 1f), while in the *WT* littermates the diaphragm extend fully anteriorly and attaches to the sternum (arrow in Fig. 1h).

Of note, the mutant mouse did not express any dorsal closure defect (Fig. 1i,k) and the closure of the palate was comparable between the knockout and the *WT* littermates (Figs 1j,l and S1i–l).

### The expression of *Tagln* in the developing embryonic organs

**Figure 2:**
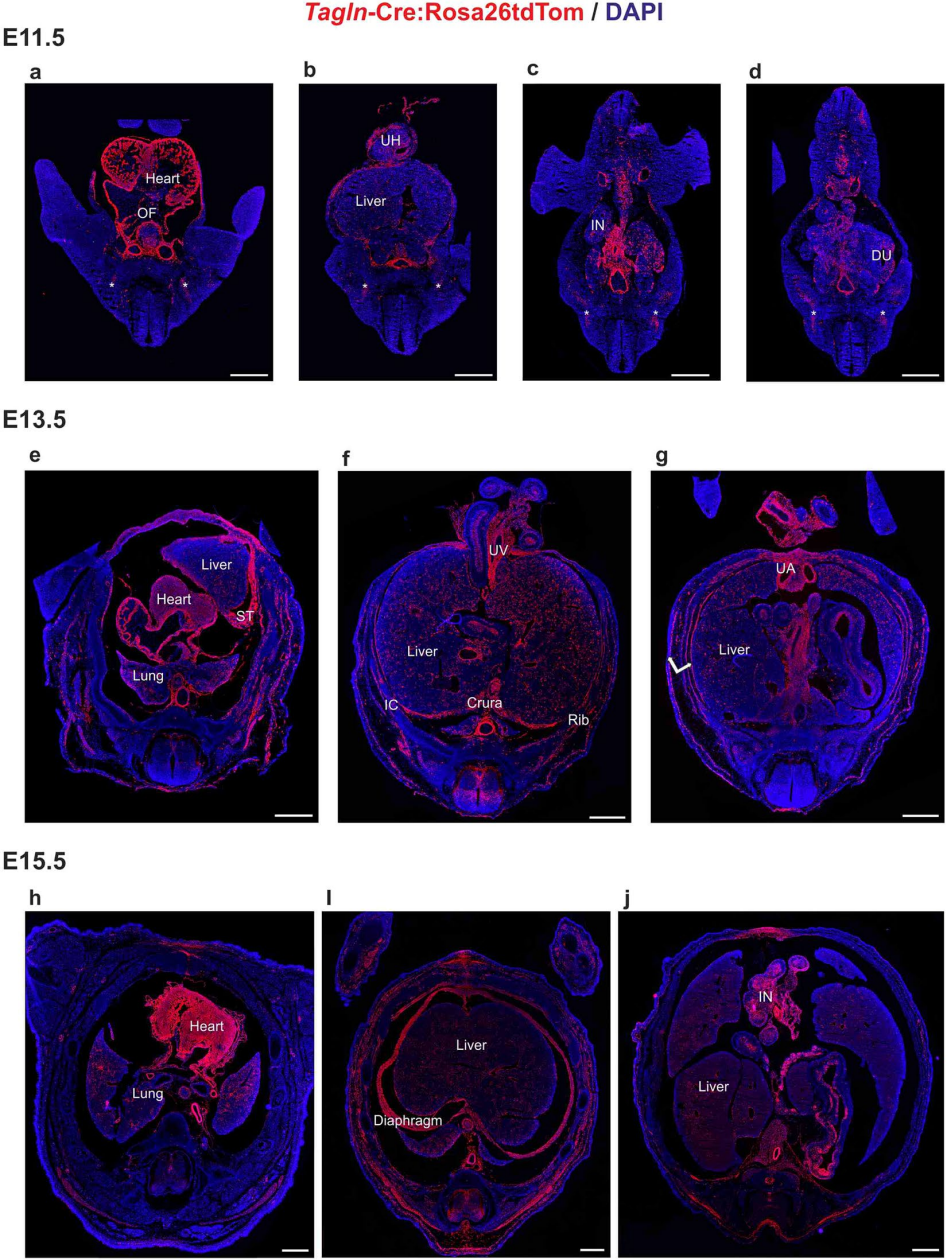
*Tagln* expression in embryonic mouse organs. *Tagln*-Cre:Rosa26-tdTomato embryo sections at E11.5, E13.5, and E15.5 stained with anti-red fluorescent protein antibody (ARFP) and DAPI. (**a**–**d**) At E11.5 tdTomato signal is evident in the developing heart, outflow tract and large vessels. The liver, intestine and the myotome (asterisk) are also showing tdTomato + cells at this stage. (**e**,**f**) At E13.5 the intercostal muscles (IC) and muscles of the abdominal wall (double arrow in **g**) are also labelled. The diaphragm is showing clear tdTomato signal both in the crural portion and in the primordial diaphragmatic tissue of the septum transversum. TdTomato signal appears in the neural tube at E13.5. (**h**–**j**) TdTomato reporter expression at E15.5 is showing a similar pattern to that of E13.5. DU: duodenum, IC: intercostal, IN: intestine, OF: outflow tract, ST: septum transversum, UA: umbilical artery, UH: umbilical hernia, UV: umbilical vein. Scale bars: 500 µm.

The 3D analysis of the knockout model enables to distinguish embryonic morphogenetic processes that depend on TGFβ signalling in *Tagln* expressing cells. *Tagln* is primarily recognized as a marker for smooth muscle cells and fibroblasts, yet a complete overview of its expression at the time of dorsal and ventral body wall closure was missing. We have recently demonstrated the *Tagln*-Cre transgene specificity and shown complete overlap between the transgene and the native TAGLN protein up until E13.5^15^. We hence used *Tagln*-Cre:Rosa26tdTom mouse model to study TAGLN expression and performed serial sectioning and IHC staining at different embryonic stages. At E11.5 *Tagln* expression is seen in the heart, outflow tract and the myotome (Fig. 2a). It also labels the aorta and the developing vasculature in diverse organs and the intestine inside and outside of the umbilical hernia (Fig. 2b–d). Remarkably, whereas *Tagln* is highly expressed in the ventral body wall, no such expression is evident in the dorsal body wall and it is absent also from the neural tube (Fig. 2a–d). At E13.5 tdTomato labelled cells are seen in many organs. In the thorax the tdTomato protein labels the heart, lungs and the major vessels (Fig. 2e). The diaphragm *crura* and the *septum transversum* are also tdTomato+ (Fig. 2e,f). Similarly, the perivascular cells in the liver and intestinal smooth muscle cells are labelled by the transgene at this stage (Fig. 2f,g). Moreover, the intercostal and abdominal muscles also express tdTomato. At E13.5 tdTomato is strongly expressed in the dorsal body wall muscles and in the interneurons of the neural tube (Fig. 2e–g). By E15.5 the expression pattern of *Tagln* is already established, the tdTomato protein expression is indistinguishable from that at E13.5 and is mainly evident in the heart, diaphragm, body wall muscles and smooth muscles of the lungs and intestine. In addition, tdTomato expression is more widely spread in the neural tube (Fig. 2h–i). These data collectively demonstrate that *Tagln* expression marks the vasculature, skeletal muscles and ventral body wall at the time of its closure, while it is initiated in dorsal structures only after the dorsal closure period.

### Diaphragmatic myogenic and myofibroblast cells express *Tagln*

**Figure 3:**
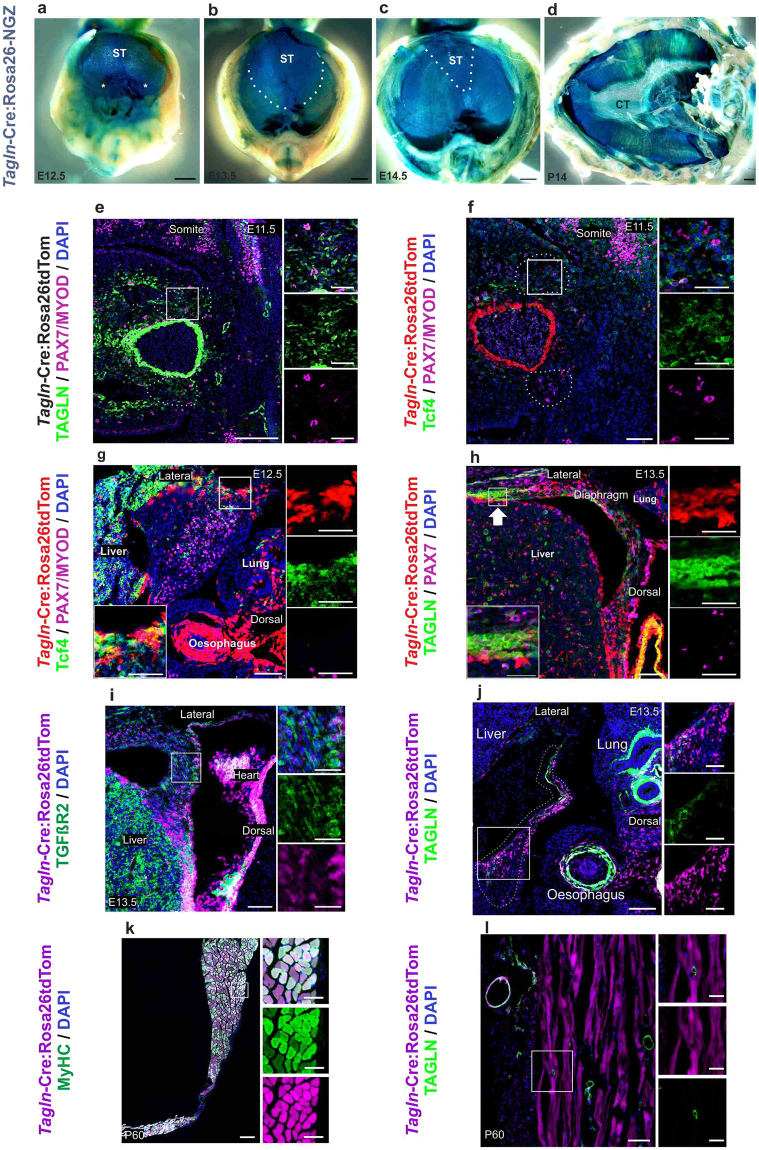
TAGLN is expressed in PPF fibroblasts and diaphragm muscle cells. (**a**–**d**) whole mount *nLacz* staining of diaphragm explant from *Tagln*-Cre:Rosa26NGZ at embryonic and postnatal stages. *Tagln*-Cre labels the PPF (asterisk in **a**), septum transversum (dotted area in **b**,**c**) and developing diaphragm muscles, while in the postnatal diaphragm this labelling is restricted to the muscular component. (**e**–**l**) Transverse sections in the PPF region of the developing embryonic diaphragm and adult diaphragm in *Tagln*-Cre:Rosa26tdTom mouse model. (**e**,**f**) The PPF at E11.5 contains TAGLN + Tcf4+ myofibroblasts and does not express signal for MyoD or Pax7 yet. (**g**) At E12.5 the tdTomato + cells are fibroblasts expressing Tcf4 and are positioned at the advancing edge of the diaphragm. While myogenic cells are located at the centre of the developing diaphragm. (**h**) At E13.5 TAGLN is upregulated at the leading edge of the developing diaphragm (arrow). (**i**) tdTomato+ cells at the leading edge of the developing diaphragm express TGFβR2. (**j**) Dorsal diaphragm cells (tdTomato+) start to downregulate TAGLN from E13.5. (**k**,**l**) Myotubes are marked by tdTomato, indicating their developmental origin from TAGLN+ myoblasts. TAGLN expression is limited to the perivascular space but not in the diaphragm muscle cells of the adult diaphragm. The dotted area in b,c represents the edges of the septum transversum and in e,f the PPF. Tagln-Cre signal in (**e**) was removed to enhance visualization of TAGLN antibody signal. CT: central tendon, ST: septum transversum. Scale bars: 500 µm in (**a**–**d**) 100 µm & insets 50 µm in (**e**–**l**).

Considering the developmental defects in the diaphragm we next focused more specifically on *Tagln* expression dynamics in the developing diaphragm. We used *Tagln*-Cre crossed to the Rosa26-NGZ reporter strain and performed whole-mount β-galactosidase staining. The expression pattern of *Tagln* from early time points of diaphragm development was remarkable in the pleuro-peritoneal folds (PPF) and the *septum transversum* (ST) (Fig. 3a). Later on during diaphragm development the muscular components, also *nLacz*+, can be seen displacing the *septum transversum* into an anterior and central position (Fig. 3b). The diaphragm continues to develop in a dorso-ventral and lateral to central fashion and the *septum transversum* is limited to a narrow triangular area at E14.5 (dotted area in Fig. 3c). In the developed diaphragm the *Tagln-*Cre derived *nLacz* signal was evident in the muscle part of the diaphragm, and to a lesser degree in the central tendon (Fig. 3d).

We next studied the cells that initiate *Tagln* expression in the developing diaphragm and constructed the cellular dynamics of the congenital defect. We analysed the developing diaphragm cells in serial sectioning and IHC staining of *Tagln*-Cre:Rosa26tdTom mouse model. At early time points of diaphragm development (E11.5) the pleuro-peritoneal folds express TAGLN, but do not express the myogenic markers paired box protien7 (PAX7) or MyoD (Fig. 3e). These early pleuro-peritoneal folds’ cells are likely fibroblasts as they co-express transcription factor 4 (Tcf4) (Fig. 3f) and TAGLN. In the PPF of E12.5 embryo tdTomato+ cells are seen in a more lateral position to the myogenic cells of the diaphragm labelled by Pax7 (Fig. 3g). These pioneering (*Tagln*+) cells at the leading edge of the developing diaphragm at E12.5 co-express fibroblast marker Tcf4, but do not express myogenic markers (Fig. 3g). This becomes more evident at E13.5 where TAGLN expression becomes increasingly restricted to the cell population at the most rostral part of the advancing diaphragm (Fig. 3h). Furthermore, tdTomato+ cells at the advancing edge of the developing diaphragm at E13.5 markedly express TGFβ receptor-2 (TGFβR2) (Fig. 3i). Starting from E13.5 some of the more dorsally-located cells of the developed part of the diaphragm start to down regulate TAGLN (Fig. 3j) while maintaining lineage tracing marker tdTomato. The TAGLN+ cells at the leading edge of the diaphragm do not appear to undergo apoptosis at time points of anterior hemi diaphragms closure and fusion (E13.5 till E15.5). We did not detect nuclear accumulation of activated Caspase 3 protein in the anterior diaphragmatic TAGLN + cells at these time points (Fig. S2a–c). The TAGLN + cells are maintained in the adult diaphragm of *Tagln*-Cre:Rosa26tdTomato as myogenic cells, pleural cells and fibroblasts of the central tendon (Figs 3k and S2d), while TAGLN expression in the adult diaphragm is only present in perivascular cells (Fig. 3l). This data demonstrates that TAGLN expressing fibroblasts arise early in PPF development and migrate ahead of the diaphragm muscle cells during diaphragm morphogenesis. It also suggests that *Tagln* is essential during diaphragm embryogenesis, but probably not for adult diaphragm maintenance.

### *Tagln*-Cre:*Tgfbr2* knockout shows anterior diaphragmatic hernia

**Figure 4:**
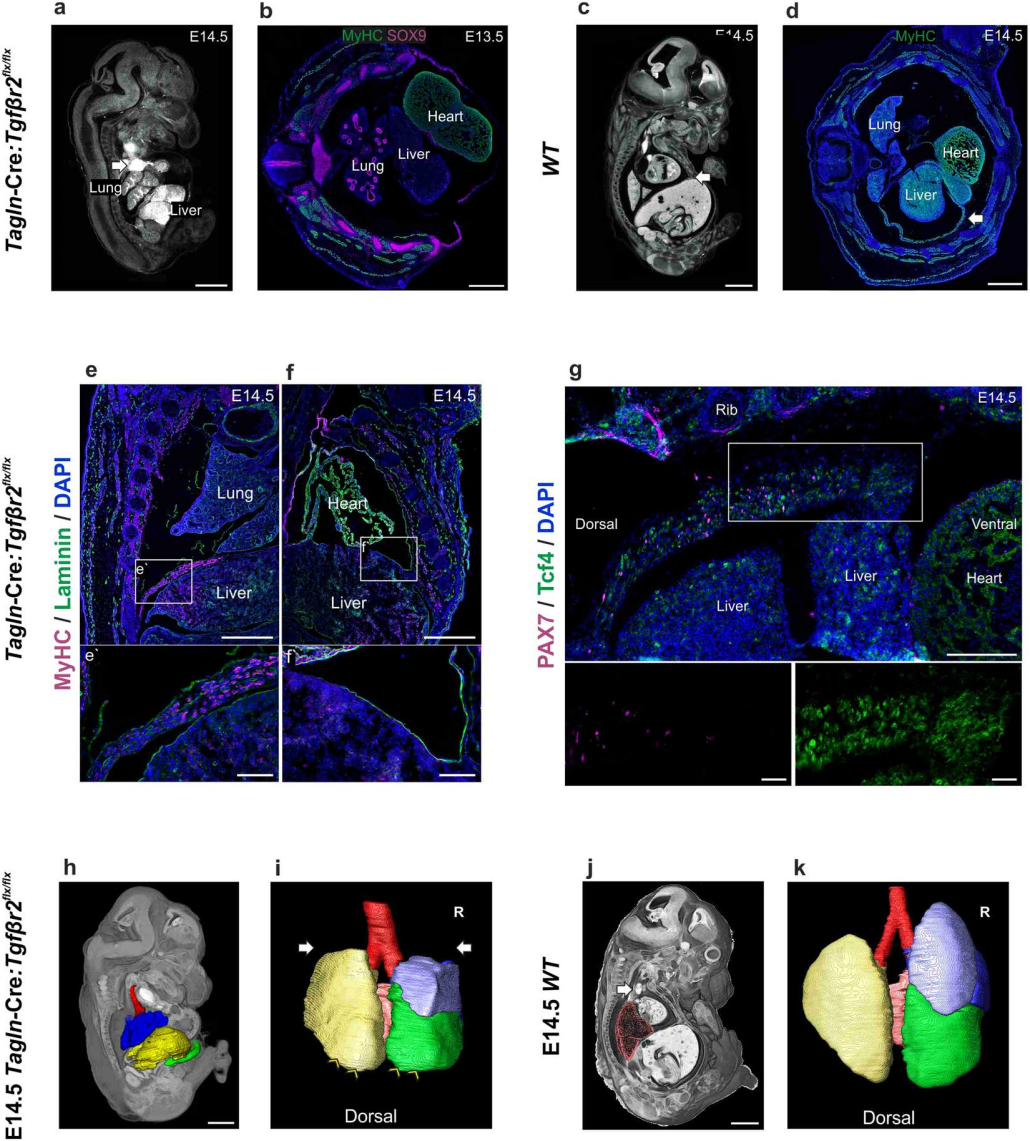
*Tagln*-Cre:*Tgfbr2* ^*flx/flx*^ shows failure of anterior diaphragm formation. (**a**,**c**) micro-CT cross sections in mutant and *WT* littermates. (**a**) the liver herniates into the chest, the dilated SVC (arrow) is seen compressing the right lung upper lobe. (**b**) transverse section in the thoracic region at E13.5 showing the liver herniating to the thoracic cavity. The lateral body wall skeletal muscles labelled by (MyHC) and rib primordia by (Sex-Determining Region Y; SOX9) are normally developed. Of note, the lung lobar configuration is maintained, left, right and posterior caval lobes are seen. (**c**) in the *WT* the diaphragm reaches the sternum (arrow). (**d**) transverse section in *WT* embryo in the thoracic region at E14.5 showing developed anterior diaphragm (arrow) made of skeletal muscle cells (MyHC+). (**e**,**f**) coronal sections to compare the posterior (**e**) and anterior (**f**) diaphragm. (**e**) the posterior diaphragm is present, extends to the lateral body wall and multiple layers of skeletal muscle cells (MyHC+) are present (**e’**). (**f**) failure of formation of a muscular anterior diaphragm and a thin sac (Laminin+) separate the abdominal and thoracic cavities (**f’**). (**g**) the leading edge of the anterior diaphragm at E14.5 fails to progress ventrally in the mutant. Condensation of Tcf4 + fibroblasts is seen, however no myogenic progenitor cells can be detected (inset). (**h**–**k**) sagittal volume rendering with segmentation of the lungs and liver in mutant and *WT* littermates. (**h**) 3D reconstruction of the liver and lungs showing the herniating liver (yellow) compressing on the lower lobes of the lungs (blue). (**i**) hypoplastic lungs in *Tagln*-Cre:*Tgfbr2* ^*flx/flx*^. The lung has normal lobar structure, however the inferior (arrowheads) and superior (arrows) lobes are smaller. (**j**) *WT* littermate showing position of liver and lung, the SVC (arrow) is positioned distant to the right lung (pink). (**k**) *WT* littermate lung. R: right. Scale bars:1,000 µm in (**a**,**c**,**h**,**j**) 500 µm in (**b**,**d**–**g**) & inset 100 µm.

We have demonstrated that TAGLN is widely expressed in the embryonic diaphragm and TGFβR2 is abundant in tdTomato+ cells at the anterior part of the developing diaphragm. In line with these observations, we found an extensive anterior diaphragmatic hernia in *Tagln*-Cre:*Tgfbr2* mutants. The anterior part of the diaphragm fails to develop completely and the liver herniates to the thoracic cavity through the anterior part of the diaphragm (Fig. 4a). We have observed the earliest evidence of this anterior diaphragmatic hernia at E13.5 when the liver can be seen herniating to the thoracic cavity displacing the heart and lungs (Fig. 4b). In the *WT* littermate, the abdominal and thoracic cavities are completely separate at E14.5. The anterior diaphragm reaches the sternum anteriorly and is made of differentiated skeletal muscle cells (Fig. 4c,d). In the mutant, by E14.5 the lateral and posterior elements of the diaphragm have developed normally and multi-layered differentiated muscle cells labelled by myosin heavy chain are seen extending between the posterio-lateral body wall and the central tendon medially (Fig. 4e,e’ and S3a [arrows]). On the other hand, the only septum between the thoracic and abdominal cavities anteriorly is a thin, Laminin positive, sac (Figs 4f,f’ and S3a [arrowheads]). Here, the most rostral portion of the developing diaphragm is made of condensated fibroblasts (Tcf4+) and lack the presence of myogenic progenitor cells (Fig. 4g). In contrast to what is seen in the *WT* at this stage where the anterior diaphragm is made of muscle cells expressing sarcomeric myosin (Fig. 4d).

The effects of liver herniation into the thorax are readily observed in the developing lungs. By using organ segmentation and volume rendering of micro-CT scans, it becomes evident that in the mutant the herniating liver pressures the lower lung lobes (Fig. 4h) compared to the *WT* littermate (Fig. 4j). The anterior parts of the lower lobes of the lungs reveal indentation from the herniating liver (Figs 4i and S3b) and are less developed than the *WT* littermates (Figs 4k and S3c). In addition, the upper lobes appear to be hypoplastic (Figs 4i and S3b) when compared to the *WT* (Figs 4k and S3c). This is probably due to direct pressure from the dilated right and left superior vena cava (Figs 4a and S3d). By using organ segmentation methods, the dilated SVCs are seen encirculating and compressing the upper lung lobes (Fig. S3e–g). The lung architecture in the mutant is otherwise maintained. Lung branching and lung lobar structure in the mutant and *WT* littermate are similar, the three right lung lobes (superior, middle and inferior), the left lobe and posterior caval lobes are all developed (Figs 4I,k and S3b,c) and the bronchial spaces are patent (Figs 4b,e and S3a). Lastly, the dilated outflow tract displaces the trachea to the left, however the tracheal lumen remains patent as in the *WT* littermates (Fig. S3h,i).

### TGF-β signalling in myogenic progenitors is not essential for diaphragm development

**Figure 5:**
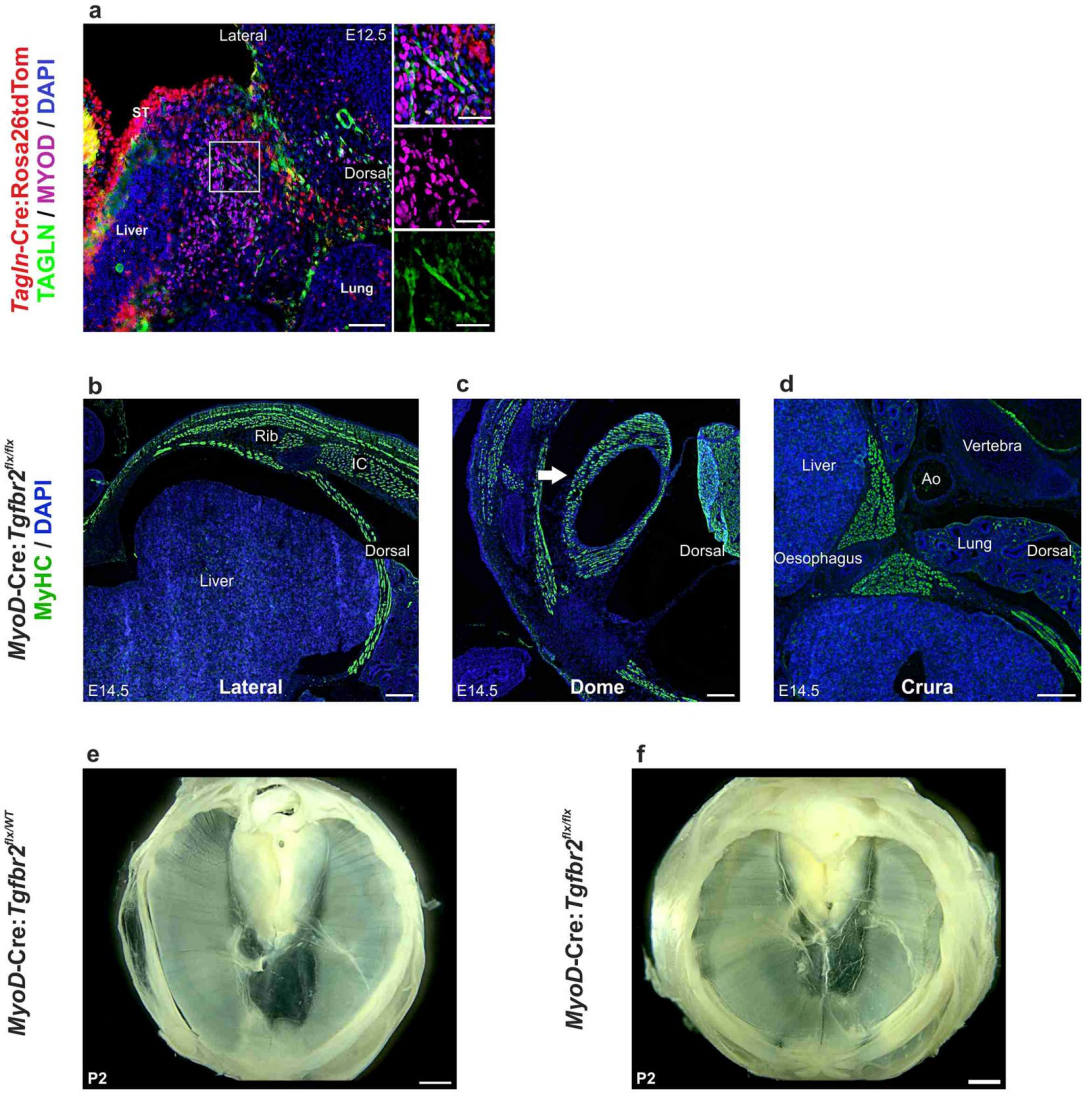
*Tgfbr2* knockout in myogenic cells does not affect diaphragm development. (**a**) transverse section in the PPF of E12.5 *Tagln*-Cre:Rosa26tdTom mouse embryo, myogenic cells (MyoD+) at this stage are present in the PPF and some of the myotubes (boxed area) express TAGLN. (**b**–**d**) transverse sections showing the different regions of the developing diaphragm in *MyoD-*Cre:*Tgfbr2*^*flx/flx*^ stained for myosin heavy chain. (**e**,**f**) diaphragm explant from *MyoD-*Cre:*Tgfbr2*^*flx/wt*^ and *MyoD-*Cre:*Tgfbr2*^*flx/flx*^ respectively showing complete diaphragmatic development. Ao: aorta, IC: intercostal muscles. Scale bars: 200 µm in (**a**–**d**) 1,000 µm in (**e**,**d**) & insets 50 µm.

The accumulating evidence suggests that fibroblast migration drives the morphogenesis of the diaphragm^30,31^. Our lineage tracing data using *Tagln*-Cre:Rosa26tdTomato model indicated that in addition to fibroblasts myogenic cells in the diaphragm undergo a phase of *Tagln* expression (Fig. 3a–c,h). This leaves open the possibility that in addition to fibroblasts myogenic cells may be affected in the *Tagln*-Cre:*Tgfbr2*^*flx/flx*^ mutant. We next analysed whether *Tgfbr2* elimination from myogenic cells may underlie the diaphragmatic development defect that we observed in *Tagln*-Cre:*Tgfbr2*^*flx/flx*^ model. We found that *Tagln* is expressed in MYOD + embryonic myotubes in the PPF from E12.5 (Fig. 5a). To characterize specifically the importance of TGFβ signalling in myogenic cells we crossed the*Tgfbr2*^*flx/flx*^ strain to the *MyoD-*Cre mouse line^32^ and analysed *MyoD-*Cre:*Tgfbr2*^*flx/flx*^ embryos and postnatal mice. Embryonic diaphragm development in *MyoD-*Cre:*Tgfbr2*^*flx/flx*^ was normal, fully muscularised diaphragmatic *crura*, dome and lateral diaphragm is present at E14.5 (Fig. 5b–d) similar to the *WT* (Figs 3c and 4d). These mice are born in good condition, do not show any signs of respiratory distress at birth and have intact and fully developed diaphragm (Fig. 5e,f). Thereby, we can conclude that TGFβ signalling during diaphragm development acts preferentially on fibroblasts and is not necessary for the patterning or development of diaphragm muscle cells.

### Cardiac and outflow tract anomalies in *Tagln*-Cre:*Tgfbr2* knockout

**Figure 6:**
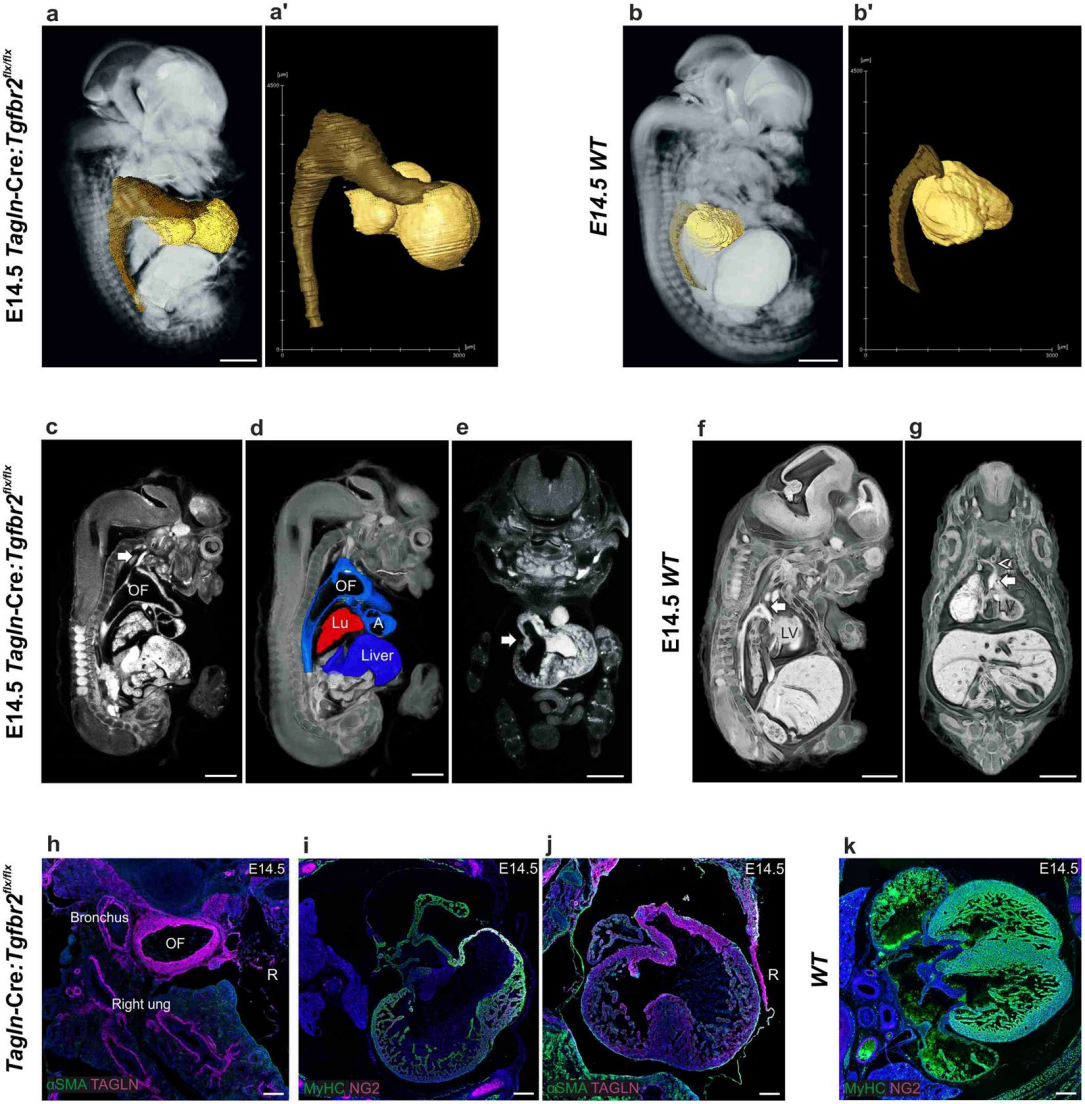
Congenital cardiac and outflow tract anomalies in *Tagln*-Cre:*Tgfbr2* ^*flx/flx*^. (**a**–**g**) micro-CT scan cross sections, volume rendering and segmentation of the heart and outflow tract in mutant and *WT* littermates. (**a**,**b**) Comparison between mutant and *WT* heart (gold) and outflow tract (bronze). Dilated aneurysmal outflow tract is evident in the mutant, extending towards the neck and filling up the superior mediastinal space. (**c**,**d**) sagittal cross sections showing the aneurysmal OF tract. (**c**) the left main carotid artery (arrow) is not dilated. (**d**) thoracic organs and liver segmentation showing the configuration of the organs in the thorax. The liver herniates cephalically and the lung occupies a small space between the OF tract and the liver. (**e**) coronal section at the level of the emerging outflow tract (arrow) showing the OF originating from the right ventricular overriding the large VSD. (**f**,**g**) sagittal and coronal sections (respectively) in a *WT* littermate embryo showing the outflow track; left sided aortic arch (arrow) is present and the emerging brachiocephalic trunk (arrowhead in g). (**h**–**k**) transverse (**i**,**k**) and coronal (**h**,**j**) sections and IHC of E14.5 *WT* and mutant embryos. (**h**) the OF tract has a TAGLN + thick wall that displaces the right main bronchus to the left. The bronchi are otherwise showing a patent lumen and a TAGLN + smooth muscle layer surrounds the main bronchi. (**i**,**j**) VSD anomaly and thin walled ventricular wall is seen. (**k**) *WT* littermates at E14.5 show complete ventricular septum. A: atrium, OF: outflow tract, Lu: lung, LV: left ventricle, R: right side. Scale bars: 1,000 µm in (**a**–**g**) 200 µm in (**h**–**k**).

Cardiac and outflow tract anomalies resulting from eliminating TGFβ signalling in *Tagln* expressing cells have been previously demonstrated^17^. However, we have characterised, for the first time, the 3D configurations of these anomalies by using micro-CT scanning. These congenital anomalies are most evident at E14.5 (Fig. 6a), both the heart and the outflow tract show gross dilatation in the mutant compared to the *WT* littermates (Fig. 6a’,b’) and the aneurysmal outflow tract occupies the majority of the superior thoracic and inferior neck spaces (Figs 6a,c,d and S4a) compared to the *WT* littermate (Fig. 6b,f). In the mutant there is a single outflow tract originating from the right ventricle and overriding the large ventricular septal defect (VSD) (Figs 6e and S4b), while the *WT* littermates at this stage show a left sided aortic arch and a separate pulmonary trunk (Fig. 6f,g). Nevertheless, in the mutant the atria and ventricles contract independently and fill the dilated outflow tract (Movie. 3). This dilated outflow tract is thick walled and displaces the right main bronchus to the left (Fig. 6h). All systemic and pulmonary branches in the mutant originate from the single outflow tract. The left and right subclavian arteries originate first, either individually directly from the outflow tract (Fig. S4b,c) or through a common arterial stem (Fig. S4d). While, the left and right common carotid arteries originate from the dome of the aneurysmal outflow tract (Fig. S4b,c,e). The pulmonary arteries originate from the posterior wall of the outflow tract origin individually and not through a common pulmonary trunk (Fig. S4c,g,h). The pulmonary veins join before draining into the left atrium (Fig. S4i,j). Of note, the aneurysmal anomaly of the arterial system is limited to the outflow tract and none of the systemic or pulmonary arteries show dilatation (Fig. S4b–h).

Structural cardiac anomalies are also found in the *Tagln*-Cre:*Tgfbr2* knockout. A large VSD is evident and the ventricular muscle wall (expressing MyHC and smooth muscle α actin (αSMA)) is thinner and less compact compared to the *WT* ventricular wall (Fig. 6i–k). These results confirms previous reports and indicates a direct requirement for TGFβ signalling in TAGLN+ vascular smooth muscle cells for the correct formation of the outflow tract and ventricular septum.

## Discussion

We provide here a comprehensive description of a mouse model for Pentalogy of Cantrell. We have used whole embryo micro-CT scanning in combination with immunohistochemistry to generate a holistic overview of the pathological changes that occur when TGFβ signalling is eliminated from *Tagln* expressing cells. This analysis provides the first mechanistic insight into the development of PC, an otherwise poorly characterised anomaly.

The phenotype expressed by the *Tagln*-Cre:*Tgfbr2* knockout resembles the phenotype seen in Pentalogy of Cantrell anomaly in humans^5^. The pentad of anomalies expressed in PC are readily observed in the mutant mouse; supra-umbilical exomphalos, ectopia cordis, anterior diaphragmatic hernia, failed formation of the diaphragmatic pericardium and intra-cardiac defects are all present. The single outflow tract overriding a VSD is normally referred to in humans as truncus arteriosus^33^. This anomaly is generally lethal and the outcome is poor^34^. This may explain the lethality seen in the *Tagln*-Cre:*Tgfbr2* ^*flx/flx*^ model. In addition, our knockout model shows gross aneurysmal dilatation of the outflow tract that has not been described in newborn infants with PC. It is likely that the lethality of this anomaly induces abortion and stillbirth and hence does not present in viable neonates. In addition, the complete loss of function nature in mouse knockout model may be the reason behind the severe anomaly spectrum in comparison to some milder anomalies in some cases of PC. Nevertheless, the resemblance between *Tagln*-Cre:*Tgfbr2* ^*flx/flx*^ and PC phenotype is quite striking.

The diverse pathologies present in PC are likely caused by morphogenetic defects mainly in the somatic mesoderm. *Tagln* is expressed in a variety of developing tissues during embryogenesis^24,35^. Beside the developing heart and outflow tract *Tagln* is seen in the myotome from as early as E9.5^24^. Moreover, *Tagln* labels migratory myofibroblasts in the developing abdominal wall^15^. We have demonstrated here that *Tagln* is abundantly expressed in the embryonic diaphragm and that TAGLN + diaphragm cells express TGFβR2. The role of TGFβ signalling in cardiac development and midline closure is widely established^14–16,36^. We have recently demonstrated a TGFβ gradient initiating from the epithelium of the primary ventral body wall (VBW)^15^. This gradient regulates the patterning of the skeletal and muscular components of the closing VBW. The peak of this TGFβ gradient is at E13.5 and the anterior diaphragmatic hernia seen in the *Tagln*-Cre:*Tgfbr2* knockout is probably a representation of a halt in diaphragmatic development at this stage. The importance of this narrow developmental window for the full closure of the diaphragm is further supported by the presence of normal posterior and lateral diaphragmatic elements in the mutant embryos. Thus, diaphragm formation is largely independent of TGFβ, whereas its morphogenetic movement depends on it. We show that both TAGLN and TGFβR2 are highly enriched in fibroblasts that accumulate at the leading edge of the closing diaphragm, making them the primary candidate to respond to the TGFβ gradient. We propose that the anterior diaphragmatic hernia seen in the *Tagln*-Cre:*Tgfbr2* ^*flx/flx*^ is probably due to dysfunction in the migration of the TGFβR2+TAGLN+cells at the leading edge of the developing diaphragm similar to what is seen in the developing body wall^15^. Importantly, we show that elimination of *Tgfbr2* from myogenic cells does not lead to any diaphragmatic defects. This data highlights the importance of TGFβ as a morphogen for controlling fibroblast-dependent tissue organization in the embryo and suggests that this may represent the key mechanism in the development of PC.

An epithelial-mesenchymal signalling is essential for the patterning and closure of the ventral body structures. Mutations in several genes involved in epithelial and mesenchymal growth result in ventral body wall closure defects. The elimination of Wnt signalling from mesenchymal cells (*Wntless* knockout in *Dermo1*Cre mouse model; *Wls*^*f/f*^*;Dermo1*^*Cre/+*^) leads to *ectopia cordis* and failure of the thoracic rib cage to close^37^. In addition, elimination of Wnt signalling in ectodermal cells (in murine msh homeobox 2 *Msx2-cre*; *Wls*^*c*/*c*^ knockout mouse model) disrupt the Wnt-Pitx 2 (Paired-like homeodomain transcription factor 2) axis, impairs ventral musculature formation and leads to ventral body wall closure defects^38^. Similarly, disruption of epithelial transcription factor AP-2 alpha (*AP2α*), aortic caroboxy-peptidase like protein (ACLP) and other components of the Wnt pathway lead to different ventral body wall closure phenotypes. For example, *Wntlss* and *β-catenin* knockout manifesting in Prune belly syndrome and *Wnt*/*β-catenin*, *Gsk-3b*, *Lrp5* and *Lrp6* knockout causing *ectopia cordis*^39–42^. We have demonstrated that the epithelium of the primary ventral body wall regulates a temporal TGFβ gradient that recruits and directs the migration of TAGLN+ fibroblasts at the leading edges of the ventral body wall and anterior diaphragm^15^. The morphological changes that shape and close the ventral body wall are dynamically controlled by multiple signalling pathways and tight cross-talk between the developing epithelial and mesenchymal components.

Micro-CT scanning has allowed for the first time complete characterisation of a multi-organ mutant mouse model. It has facilitated displaying the full picture and enabled visualization of smaller defects and anatomical variations that are otherwise difficult to establish by simple histological analysis of sectioned specimens. The micro-CT scanning method we have used allowed us to visualise sections at 6 µm intervals without the risk of losing any section that is commonly encountered in standard histological analyses. In addition, the use of contrast staining methods allows staining soft tissues differentially and hence acquiring images with high inter-tissue resolution. Furthermore, the ability to generate different angle views and off-axis sections provides 3-dimensional overview of the development of the pathology.

We have demonstrated in this study that TGFβ signalling in *Tagln* expressing cells displays a significant role in the development of mesodermal tissues and the loss of this role manifests in embryonic defects that are highly similar to the Pentalogy of Cantrell.

## Materials and Methods

### Animals

Mice were housed and bread in the University of Manchester animal facility. Mouse models have been published previously: *Tagln-*Cre^24^, *MyoD-*Cre^32^ and *Tgfbr2*^*flx/wt*^ ^43^. *Tagln*-Cre mice were crossed with C57BL Rosa26 tdTomato^44^ and CD1 Rosa NGZ/ LacZ [Gt(ROSA)26Sortm1(CAG-lacZ,-EGFP)Glh]^45^ reporter mice. *Tagln-*Cre*:Tgfbr2* ^*flx/flx*^ and *MyoD-*Cre:*Tgfbr2*^*flx/flx*^ were obtained by crossing the *Tagln-*Cre and *MyoD-*Cre, respectively, to *Tgfbr2*^*flx/wt*^ mice and the off spring was crossbred to obtain homozygous embryos confirmed by genotyping. All animal work was conducted according to the Home Office regulations and was approved under license no. 707435.

### Immunofluorescence staining

Embryos were fixed in 4% paraformaldehyde (PFA) in PBS solution for 24 hours. The embryos were washed thoroughly with PBS and dehydrated in a sucrose gradient overnight. Embryos were embedded in an OCT mould (Clinipath) and snap-frozen in liquid nitrogen. Cryosections (7–10 μm) were cut using Leica cryostat (CM3050). Slides were washed in PBS, PBS with 0.2% tween 20 (3 times 5 minutes) and then blocked in an incubation buffer (10% normal donkey serum, 1% bovine serum albumin (BSA) and 0.2% tween 20 in PBS) for 4 hours. Primary antibody was added and incubated overnight at 4 °C. On day 2 the slides were washed with 0.2% PBS-Tween 20 and blocked with a second incubation buffer (1% BSA and 0.2% tween 20 in PBS) for 1 hour at room temperature. Secondary antibodies specific to the primary antibody host species was added and incubated for 1 hour at room temperature. Slides were washed and mounted in Vectashield mounting media with DAPI (Vectalabs). Slides were imaged using Zeiss Axio Imager M2. Zeiss Zen software was used for image analysis.

### Whole mount IHC

Whole mount IHC were described elsewhere^30^. Briefly, embryos were fixed in 4% PFA for 24 hours, washed with PBS, bleached for 24 h with Dent’s bleach, rinsed with 100% methanol and then fixed in Dent’s fix. Specimens were placed in Dent’s fixed for at least one week prior to incubation with primary antibody. Specimens were then washed with PBS, blocked in 5% donkey serum in PBS and incubated with primary antibody(s) for 72 hours. Specimens were washed with PBS thoroughly and incubated with secondary antibody(s) for 72 hours. Specimens were then rinsed in methanol, methanol-BABB and cleared in 100% BABB solution. Specimens were then placed in a silicone container, covered in BABB and imaged using two photon laser confocal microscope (Leica SP8 Upright). Three-dimensional data sets were analysed with Leica confocal and Bitplane/ Imaris software.

Solutions:

Dent’s fixative: 1 part DMSO: 4 parts Methanol

Dent’s Bleach: 1 part H2O2: 2 parts Dent’s fix

BABB: 1 part benzyl alcohol: 2 parts benzyl benzoate

Blocking Serum: 5% donkey serum (also from Jackson Labs), 75% 1xPBS, 20% DMSO

### Antibodies

A full list of primary antibodies used in this work can be found in supplementary table (1). Diverse secondary antibodies were used in this study (lifetech).

### Whole mount β -gal staining in diaphragm explants

Diaphragm explants were generated from fixed embryos (as above). The Torsos was dissected to include the lower thorax with the upper abdomen regions. The heart and lungs were removed carefully not to damage the diaphragm beneath and the liver was left en-bloc with the diaphragm explant. Diaphragm explants were permeabilised in (1% Triton X and 0.4% NP40 in PBS) solution for 4 hours and incubated overnight for β-galactosidase activity at 37 °C as described^46^. Embryos were imaged using Zeiss Axio Zoom microscope and Zeiss Zen software was used for image analysis.

### Micro-CT scans

#### Tissue treatment and staining

Embryos of desired gestation were collected fresh and fixed for 24 hours in 4% PFA solution. They were rinsed thoroughly in PBS and placed in PBS 10% sucrose for 4–6 hours to avoid shrinkage when adding staining (contrast) solution. The staining method was adapted from^47,48^. The staining solution is made of 1 part Lugol’s solution and 3 parts iodine free water with 10% sucrose. Samples were incubated in staining solution for 48–72 hours at 4 °c on a rocking surface and protected from light. Solutions:

Lugol’s solution (Make fresh):

10 g KI (Potassium iodine) in 100 mls H2O when dissociates add

5 g I2 (elemental iodine) [protect from direct light]

Staining solution (25% Lugol’s in 7% sucrose):

1 part 100% Lugol’s to 3 parts iodine free water 10% sucrose

#### Micro-CT acquisition

Micro-CT data acquisitions were performed using a Nikon XTH 225 kV instrument with a tungsten target. Acquisition parameters were chosen to optimise absorption contrast and were 80 kV and 180 µA, without using a filter in the beam path. Around 5000 projections with an exposure time of 500 ms were taken per scan. Each specimen was scanned individually to achieve a voxel size of 6 µm x 6 µm x 6 µm. The resulting data were reconstructed using Nikon 3D Pro reconstruction software, before exporting for segmentation and visualisation in FEI VSG Avizo software. All CT scan images comparing mutant and *WT* embryos were performed on littermate embryos (from the same conception) after confirmation with genotyping.

#### Ethical approval

All animal work in this study was conducted according to the Home Office regulations and was approved by the Home Office under license number 70/7435 according to the UK Animals (Scientific Procedures) Act (1986). The University of Manchester Research Ethics Review board has approved this study.

### Data availability

The methodology of all experiments performed is fully described in the methods section. All materials and mouse models used can be obtained from the corresponding author upon request.

## Electronic supplementary material

Movie one

Movie two

Movie three

Supplementary info
