# Supplementary material for: Abrogation of TGF-beta signalling in TAGLN expressing cells recapitulates Pentalogy of Cantrell in the mouse
Source: Sci Rep. 2018 Feb 26;8:3658. doi: 10.1038/s41598-018-21948-z (PMC5826924; doi:10.1038/s41598-018-21948-z)

Abrogation of TGF-beta signalling in TAGLN expressing cells recapitulates Pentalogy of Cantrell in the mouse

Bashar Aldeiri[1-2*], Urmas Roostalu[1], Alessandra Albertini[5], Julia Behnsen[4], Jason Wong[1-3], Antonino Morabito[1-2] and Giulio Cossu[1]

Supplementary information:

Supplementary figures legend:

Figure supplemental (1): Congenital anomalies in *Tagln*-Cre:*Tgfbr2* $^{flx/flx}$. a-b: micro-CT volume rendering and organ segmentation showing physiological umbilical hernia (arrow in a) in the *WT* while the mutant (b) has a gross ventral body wall closure defect with ectopia cordis (blue) and exomphalos (green and the umbilical cord [arrow] marked in a dashed line) at E14.5. c-d: whole mount IHC 3D reconstruction showing normal ventral closure and physiological umbilical hernia (arrow in c) at E14.5 *WT* embryo. In the *Tagln*-Cre:*Tgfbr2* $^{flx/flx}$ a Laminin positive thin sac covers the ventral body wall. An exomphalos major containing the liver and intestine (Vimentin positive) is evident and ectopia cordis (arrowhead in d) is seen. e-h: sagittal 3D reconstruction of whole mount IHC using Laminin and Vimentin antibodies. e: the mesenchymal lateral body wall components fail to advance ventrally (arrowheads) and a Laminin positive thin sac is seen covering the ventral organs. f: Aneurysm is evident in the outflow tract (arrows), whereas the distal descending and abdominal aorta have largely normal morphology (arrowheads). g: Both internal jugular and subclavian veins are dilated (arrow). h: rupture lines are seen in the subclavian vein (arrow) and superior vena cava (SVC). i-l: Off-axis sections to show the palate. Normal palate developmental milestones in the mutant and *WT* at E14.5. Fusion of the anterior (i) but not the posterior (j) part of the palate (arrows) in the mutant that is comparable to what is seen in the *WT* littermates (k-l). A: atria, IVC: inferior vena cava, OF: outflow tract, SVC: superior vena cava, V: ventricle. Scale bars: 1,000 µm.

Figure supplemental (2): The fate of diaphragm TAGLN+ cells in the *Tagln*-Cre mouse model. a-c: Transverse sections in the anterior diaphragm of *Tagln*-Cre:Rosa26tdTom at E13.5 to E15.5 does not show increased Caspase 3 activity of the TAGLN+ cells at the leading edge of the developing diaphragm (E13.5 – E14.5) or at time of anterior hemi diaphragms fusion (E15.5). Some scattered Caspase 3+ cells are seen (arrows). d: transverse section of a hemi diaphragm (between the crura [bottom left] and lateral diaphragm [top right]) of adult *Tagln*-Cre:Rosa26tdTom showing the muscular portions and the central tendon. d’: magnified view of the muscular diaphragm, tdTomato signal is present in the muscle fibres, pleura (αSMA+, Laminin+; arrowhead) and fibroblasts (αSMA+, arrow). d’’: magnified view of the central tendon, tdTomato signal is present in the pleural lining (arrowhead) and fibroblasts (arrow). Scale bars: 200 µm & insets 50 µm.

Figure supplemental (3): Congenital anomalies of thoracic organs in *Tagln*-Cre:*Tgfbr2* $^{flx/flx}$. a: transverse section in the thoracic region at E13.5 showing developed posterior-lateral diaphragm (arrows) and failure of anterior diaphragm development (arrowheads). b,c: lung segmentation of mutant and *WT* littermates*.* The lung is still made of a left lobe (yellow), posterior caval lobe (pink) and three right lobes; superior (violet), middle (blue) and inferior (green). However, the upper and lower lobes are hypoplastic in the mutant. d: coronal section in the mutant showing the two SVCs (arrows) pressuring the superior lung lobes. An asterisk labels the dilated outflow tract. e-g: segmentation of the lung (light blue) and SVC (red) demonstrating the dilated veins wrap around and pressure the superior lung lobes. h,i: coronal sections showing the dilated OF tract (asterisk) displacing the trachea (arrow) to the left in the mutant (h) and the normal configuration in *WT* littermate (i). Nevertheless, the tracheal lumen remains patent. Lu: lung, R: right side. Scale bars: 1,000 µm except 500 µm in a.

Figure supplemental (4): Major branches of the OF tract in *Tagln*-Cre:*Tgfbr2* $^{flx/flx}$. a: micro-CT volume rendering with segmented heart (blue) and outflow tract (red) in the *Tagln*-Cre:*Tgfbr2* $^{flx/flx}$ mutant embryo. The serial sagittal sections show the gross dilatation of the OF tract aneurysm that otherwise spares the distal descending and abdominal aorta. The serial sections start from the left side of the embryo and continue towards the right (left to right on panel). b,c: segmentation of the heart (bronze), OF tract (shaded), descending aorta (gold), vena cava (brown) and major branches (dark grey). OF tract originates from the right ventricle and gives rise to the major arteries. The left SVC is removed from the lateral view to show the origin of the pulmonary arteries. d-j: transverse micro-CT sections showing the major branches of the OF tract (the right side is to the top of the panel). d; the subclavian arteries (arrows) originates from the OF via a common trunk. e: The common carotid arteries (arrows) originates in the neck from the dome of the OF. f: The carotid arteries in the neck are not dilated, the left common carotid has divided into the internal and external branches. g,h: the pulmonary arteries (arrow) originates individually from the posterior wall of the OF and travel towards the main bronchus (arrowhead). i,j: The pulmonary veins join to form a common trunk (arrow in i) before draining into the left atrium (arrow in j). AO: aorta, CCA: common carotid artery, d.AO: descending aorta, LA: left atrium, LSCA: left subclavian artery, LSCV: left subclavian vein, Lu: lung, LV: left ventricle, OF: outflow tract, PA: pulmonary artery, RA: right atrium, RSCA: right subclavian artery, RSCV: right subclavian vein, RV: right ventricle. Scale bars: 500 µm.

Supplemental Movie 1: 3D reconstruction of whole mount IHC in an E14.5 *Tagln*-Cre:*Tgfbr2* $^{flx/flx}$ embryo. Complete ventral body wall closure defect, only a thin sac (Laminin+) covers the mesenchymal (Vimentin+) ventral organs.

Supplemental Movie 2: 3D reconstruction of whole mount IHC in an E14.5 *Tagln*-Cre:Rosa26tdTom embryo. The ventral body wall only shows a physiological umbilical hernia at this stage of development.

Supplemental Movie 3: Freshly harvested mutant embryo at E14.5. Complete failure of ventral body wall closure and a thin sac covers the ventral organs. Synchronous atrial and ventricular contractions are still present. Two ventricular cavities can be seen at end systole and the ventricular contraction fills in the dilated outflow tract. Movie played at five frames per second.

Table (1) Primary antibodies used:

Antibody	Host	Source	Conce-ntration	Product No.	Lot No.	Dilution
TAGLN (αSM22)	Rabbit	Abcam	1 mg/ml	AB14106	GR274712-2	1:100
TAGLN (αSM22)	Goat	Abcam	0.5mg/ml	AB10135	GR1926-4	1:50
αSMA	Goat	Abcam	0.5 mg/ml	AB 21027	GR230733-4	1:200
NG2	Rabbit	Millipore	100 ug	AB 5320	2669913	1:300
TGFβR2	Rabbit	Novus Bio	0.1 mg/ml	NB100-91994	GN1151	1:100
RFP	Rabbit	Rockland	100 ug	600-401-379	34944	1:500
Vimentin	Chicken	Novus Bio	-	NB300-223	1-0104	1:300
MyHC	Mouse	DSHB	-	AB247781	-	1:3
PAX7	Mouse	DSHB	-	AB2147781		1:2
MyoD	Mouse	Dako	0.5 mg/ml	M3512	10079010	1:100
TCF4	Rabbit	Abcam	1.91 mg/ml	Ab185736	GR3172982	1:50
Caspase-3	Rabbit	Abcam	0.5 mg/ml	Ab13847	Gr300156-1	1:100

Tcf4 reaction required an amplification step using biotin-conjugated donkey anti rabbit (Jackson immuno Research) followed by streptavidin conjugated Dylight488 (Jackson immuno Research) as described [1].

Supplementary references:

1. Merrell, A. J. *et al.* Muscle connective tissue controls development of the diaphragm and is a source of congenital diaphragmatic hernias. *Nat. Genet.* **47,** 496–504 (2015).

Figure supplemental 1

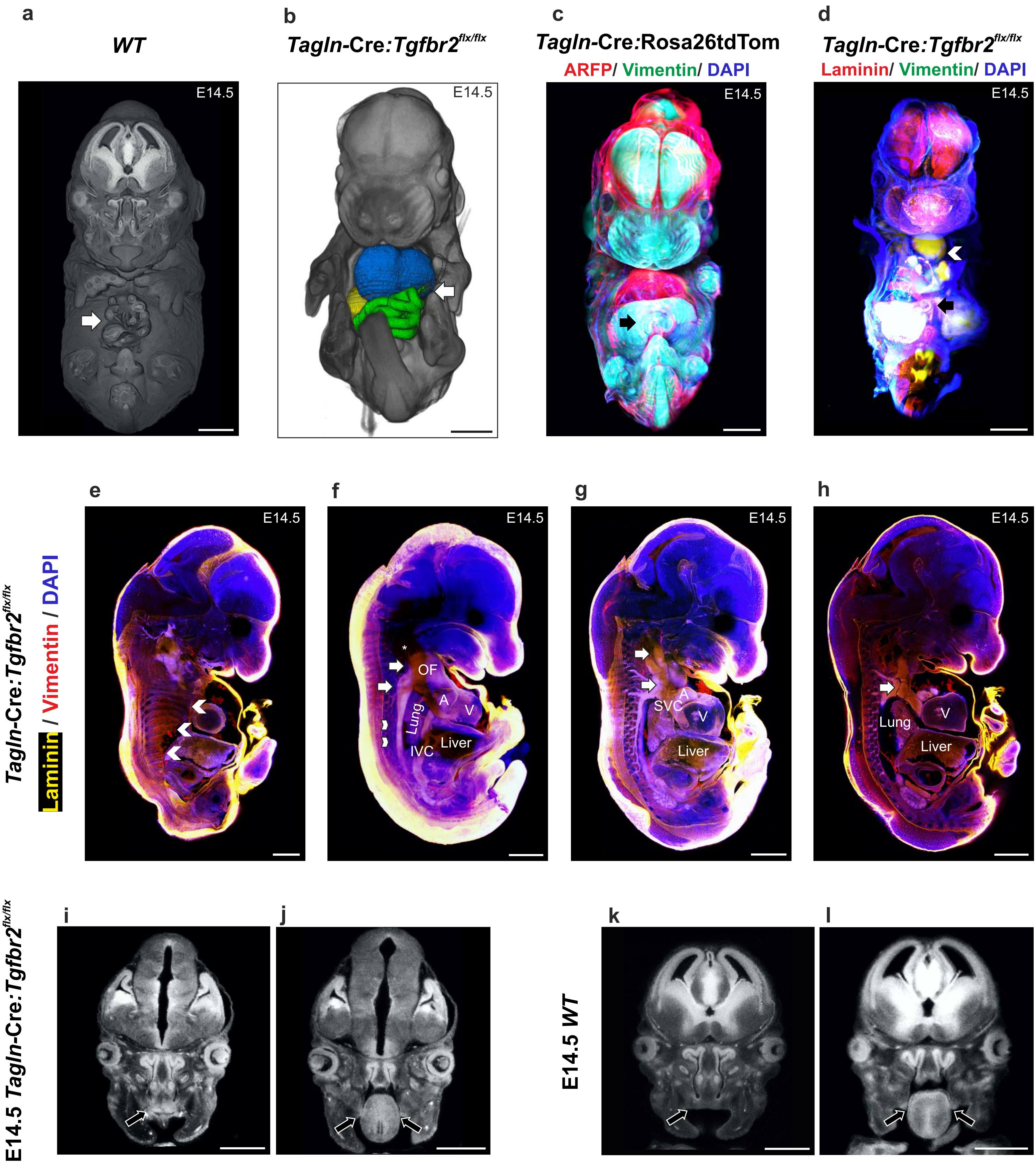

Figure supplemental 2

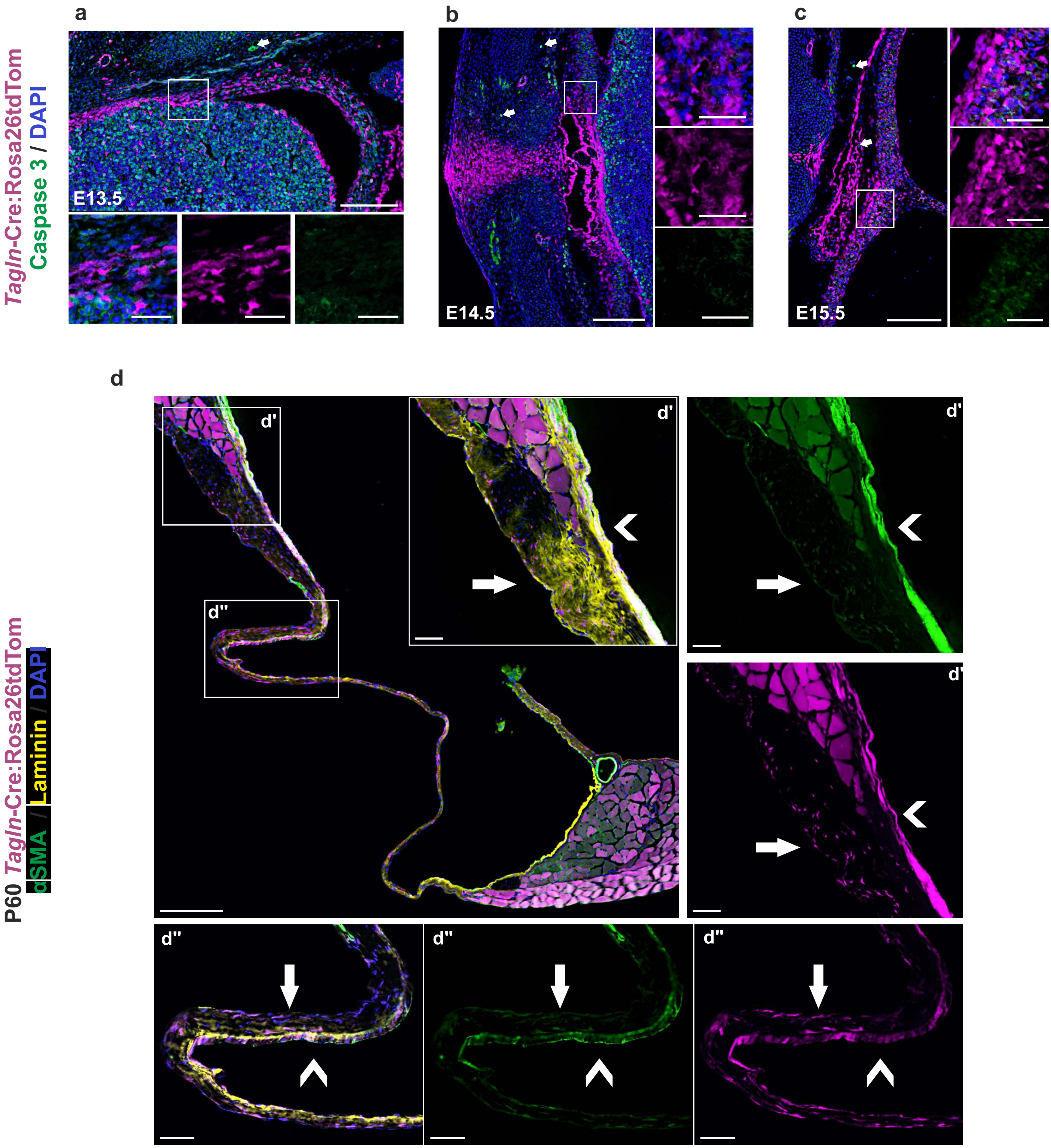

Figure supplemental 3

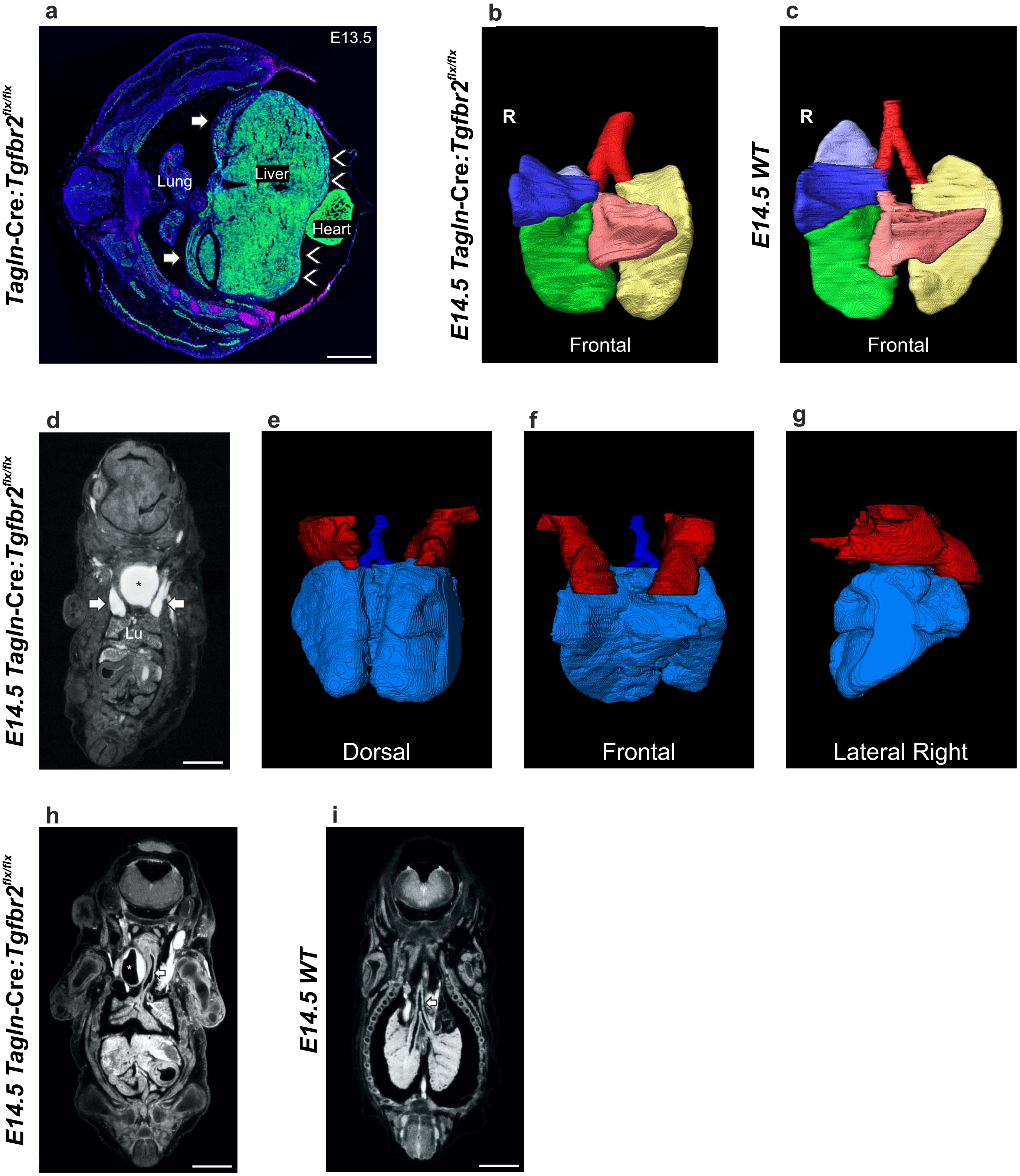

Figure supplemental 4

E14.5 *Tagln*-Cre:*Tgfbr2*$^{flx/flx}$

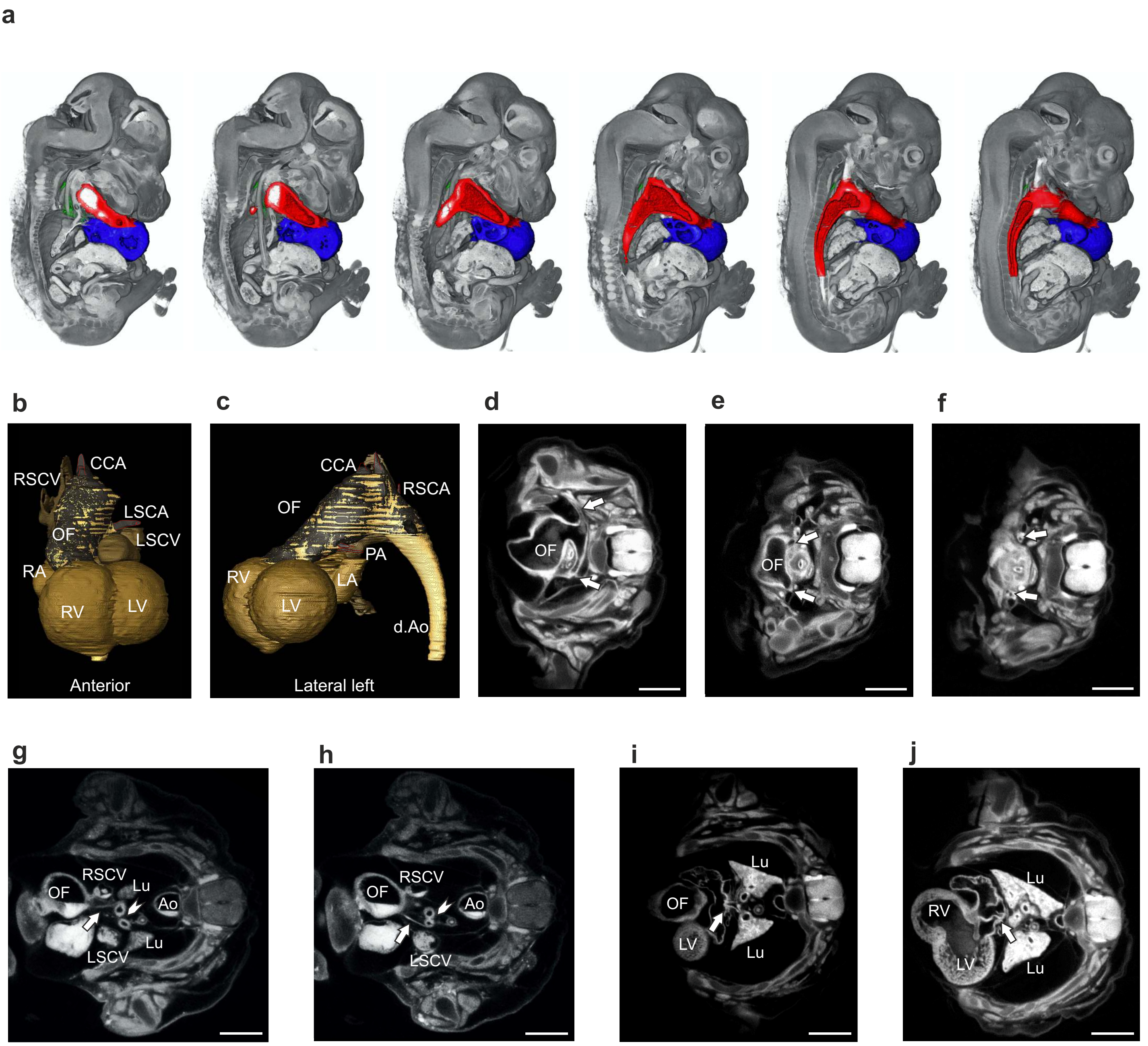

Supplement: Supplementary file 4 — Supplementary info [file 41598_2018_21948_MOESM4_ESM.pdf]
